# Evaluating the Effect of Labeled Benchmarks on Children’s Number Line Estimation Performance and Strategy Use

**DOI:** 10.3389/fpsyg.2017.01082

**Published:** 2017-06-30

**Authors:** Dominique Peeters, Elke Sekeris, Lieven Verschaffel, Koen Luwel

**Affiliations:** ^1^Psychology and Educational Sciences, KU LeuvenLeuven, Belgium; ^2^KU Leuven – Campus BrusselsBrussels, Belgium

**Keywords:** number line estimation, strategy use, benchmark support, quartile-based strategies, verbal protocols

## Abstract

Some authors argue that age-related improvements in number line estimation (NLE) performance result from changes in strategy use. More specifically, children’s strategy use develops from only using the origin of the number line, to using the origin and the endpoint, to eventually also relying on the midpoint of the number line. Recently, Peeters et al. (unpublished) investigated whether the provision of additional unlabeled benchmarks at 25, 50, and 75% of the number line, positively affects third and fifth graders’ NLE performance and benchmark-based strategy use. It was found that only the older children benefitted from the presence of these benchmarks at the quartiles of the number line (i.e., 25 and 75%), as they made more use of these benchmarks, leading to more accurate estimates. A possible explanation for this lack of improvement in third graders might be their inability to correctly link the presented benchmarks with their corresponding numerical values. In the present study, we investigated whether labeling these benchmarks with their corresponding numerical values, would have a positive effect on younger children’s NLE performance and quartile-based strategy use as well. Third and sixth graders were assigned to one of three conditions: (a) a *control* condition with an empty number line bounded by 0 at the origin and 1,000 at the endpoint, (b) an *unlabeled* condition with three additional external benchmarks without numerical labels at 25, 50, and 75% of the number line, and (c) a *labeled* condition in which these benchmarks were labeled with 250, 500, and 750, respectively. Results indicated that labeling the benchmarks has a positive effect on third graders’ NLE performance and quartile-based strategy use, whereas sixth graders already benefited from the mere provision of unlabeled benchmarks. These findings imply that children’s benchmark-based strategy use can be stimulated by adding additional externally provided benchmarks on the number line, but that, depending on children’s age and familiarity with the number range, these additional external benchmarks might need to be labeled.

## Introduction

The number line estimation (NLE) task has been extensively investigated during the last decade. In this task, participants are asked to estimate the spatial position of a number on an empty number line with labeled endpoints (e.g., 0 and 1,000). Developmental studies have shown age-related improvements in estimation performance (e.g., Siegler and Booth, 2004; Booth and Siegler, 2006, 2008; Berteletti et al., 2010). According to the log-to-lin shift hypothesis put forward by Siegler and Opfer (2003), these improvements can be explained by changes in children’s mental magnitude representation of a particular number range, which is assumed to gradually shift from a logarithmic toward a linear representation when children get more acquainted with this range (e.g., Siegler and Booth, 2004; Booth and Siegler, 2006; Laski and Siegler, 2007).

Recently, some researchers have argued that these developmental changes in NLE performance might be the result of an increasing use of more sophisticated benchmarks instead of a shift in the underlying numerical magnitude representation (e.g., Barth and Paladino, 2011; Cohen and Blanc-Goldhammer, 2011; Ashcraft and Moore, 2012; White and Szucs, 2012; Slusser et al., 2013; Link et al., 2014a; Rouder and Geary, 2014; Peeters et al., 2016, 2017, unpublished). Evidence in favor of this benchmark use is based on several sources, such as participants’ error rates and estimation latencies (Ashcraft and Moore, 2012), the superior fittings of one- and two-cycle power functions compared to logarithmic and linear functions on individuals’ estimation patterns (Barth and Paladino, 2011; Cohen and Blanc-Goldhammer, 2011; Slusser et al., 2013; Rouder and Geary, 2014; Barth et al., 2015; Reinert et al., 2015, though see Opfer et al., 2016), verbal reports of participants’ solution behavior (Newman and Berger, 1984; Peeters et al., 2016, 2017, unpublished), and, finally, eye-movement data (Schneider et al., 2008; Heine et al., 2010; Sullivan et al., 2011). Based on the evidence coming from all these sources, the development in children’s benchmark use can be depicted as follows. Initially, children only rely on the externally provided benchmark (or briefly: external benchmark) at the origin of the number line for estimating all target numbers. Next, they use the origin to estimate target numbers in the lower range and the endpoint to position target numbers in the upper range of the number line. Later on, children generate a third, self-derived benchmark (or briefly: internal benchmark) at the midpoint for locating target numbers in the middle range of the number line (Newman and Berger, 1984; Petitto, 1990; Schneider et al., 2008; Barth and Paladino, 2011; Ashcraft and Moore, 2012; Slusser et al., 2013; Xu and LeFevre, 2016).

Recently, Peeters et al. (2017) demonstrated that the evolution in the application of benchmark-based strategies does not stop with the creation and use of an internal benchmark at the midpoint of the number line. Almost all participants in their adult sample generated even more refined internal benchmarks at the quartiles (i.e., 25 and 75%) of a 0–1,000 number line. In a follow-up study, Peeters et al. (unpublished) investigated the extent to which third and fifth grade children are also able to use the quartiles on a 0–1,000 number line, by varying the degree of provided benchmark support on the number line. This support was manipulated at three levels. In the control condition only the origin and endpoint were indicated by two external benchmarks, in the midpoint condition one additional external benchmark at 50% was provided, while in the quartile condition three additional external benchmarks at 25, 50, and 75% were given. No numerical labels were given for these additional external benchmarks, so children had to derive the corresponding numerical value of these benchmarks themselves. It was found that some third graders already created quartile benchmarks in the control condition and that by fifth grade about a quarter of the children were capable of generating and using the two additional internal benchmarks at 25 and 75% of the number line. Results further indicated that the provision of additional external benchmarks led to more accurate estimates and stimulated the use of quartile-based strategies in fifth graders. Third graders, on the other hand, seemed to benefit less from the given support, as they estimated about equally accurately in all three conditions and resorted more to (less advanced) idiosyncratic strategies, especially when all three additional external benchmarks were provided. Peeters et al. (unpublished) also found that relying on a larger variety of benchmark-based strategies and applying these strategies more frequently was positively related to estimation accuracy and children’s mathematics achievement.

Peeters et al. (unpublished) explained third graders’ lack in increase of quartile-based strategies in the quartile condition by their inability to (correctly) link the additional external benchmarks at 25, 50, and 75% with their corresponding numerical values. This might have been due to the fact that, in contrast to fifth graders, they were not yet sufficiently familiar with the presented number range. If this explanation is correct, then providing the numerical values of the respective benchmarks on the number line should have a beneficial effect on children’s quartile-based strategy use and, thus, on their estimation performance. Hence, the main aim of the present study was to examine whether the provision of *labeled* external benchmarks at 25, 50, and 75% would have a positive effect on third and sixth graders’ estimation performance and quartile-based strategy use on a 0–1,000 number line and whether this effect differs with age.

We compared children’s performance and strategy use in three different conditions. In the control condition, children were presented with an empty number line bounded by 0 at the origin and 1,000 at the endpoint. In the unlabeled condition, three additional external benchmarks without numerical labels were given at 25, 50, and 75% of the number line. Finally, children in the labeled condition were given the same external benchmarks as in the unlabeled condition but now together with their corresponding numerical labels (i.e., 250, 500, and 750).

Moreover, we examined whether the potential positive effect of labeling was dependent on children’s age by comparing third to sixth graders’ estimation performance and strategy use.

Taking into account the findings of Peeters et al. (2017, unpublished), the following two hypotheses were raised. First, we hypothesized that the mere provision of the unlabeled benchmarks at 25, 50, and 75% of the number line would have a positive effect on sixth graders’ estimation performance and strategy use, but that the labeling of the benchmarks itself would have no additional beneficial effect. The reason is that children in this age group are fully familiar with the 0–1,000 number range, which enables them to link the provided external benchmarks with their numerical values. Moreover, Peeters et al. (unpublished) found that even fifth graders were already able to correctly identify and use the presented unlabeled benchmarks at 25, 50, and 75% of the number line. We therefore expected that sixth graders would estimate more accurately, use a larger variety of benchmark-based strategies, and apply the quartile-based strategies more frequently in the unlabeled and labeled condition in comparison to the control condition. Based on the above-mentioned findings in third graders by Peeters et al. (unpublished) and because the 0–1,000 range is less familiar to third graders, we expected that the mere presentation of additional unlabeled benchmarks would not suffice to positively affect third graders’ estimation performance and strategy use, but that an additional labeling of the benchmarks would be needed to obtain such a beneficial effect. Hence, we predicted that third graders’ estimation accuracy, variety in benchmark-based strategies, and frequency of use of quartile-based strategies would be larger in the labeled than in the control and unlabeled condition.

Finally, in line with Peeters et al. (unpublished), we expected that, for both grades, a larger variety of benchmark-based strategies and a higher frequency in the use of quartile-based strategies would be positively related to children’s NLE accuracy and to their general mathematics achievement.

## Materials and Methods

### Participants

Eighty-three third graders (*M* = 8 years, *SD* = 0.46 years, 43 boys) and 72 sixth graders (*M* = 11 years, *SD* = 0.50 years, 36 boys) from two elementary schools located in the rural area of Flanders, Belgium, participated in the experiment. The experiment was conducted according to the institutional ethical guidelines at that time. The teachers and principals gave permission for the experiment, the parents agreed through informed consent. Children were told that they could quit the experiment at any moment.

### Materials and Procedure

Children’s NLE performance was assessed by a number-to-position task with a number line labeled by 0 at the origin and by 1,000 at the endpoint. For each target number, a new number line with a length of 23.5 cm was presented on a separate sheet of paper. To avoid that the position of the target number might function as an (unintended) external benchmark (Dackermann et al., 2015), it was presented on a separate card that was placed on the table in front of the child at a randomly selected position.

**FIGURE 1:**
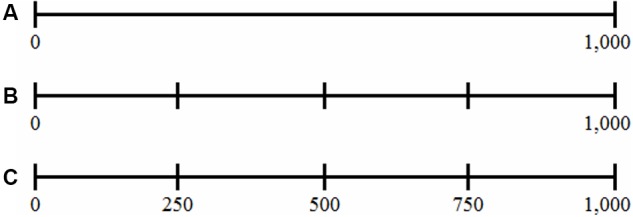
Presented number line in the control **(A)**, unlabeled **(B)**, and labeled **(C)** condition.

Children were randomly assigned to one of three conditions – a control condition, an unlabeled condition, and a labeled condition – with the following restrictions: (a) children were matched across these conditions with respect to their mathematics achievement scores, and (b) the number of boys and girls was distributed equally across conditions. In the control condition (**Figure 1A**), children were presented with an empty number line bounded by two labeled external benchmarks (i.e., the origin and the endpoint) indicated by a small hatch mark and their corresponding numbers (i.e., 0 and 1,000) were printed underneath these hatch marks. In the unlabeled condition (**Figure 1B**), the number line contained three additional external benchmarks, indicated by hatch marks at 25, 50, and 75% of the number line. In the labeled condition, the hatch marks at 25, 50, and 75% of the number line were labeled (**Figure 1C**), by presenting their corresponding number (i.e., 250, 500, and 750, respectively) underneath each mark.

The NLE task was administered individually in a quiet room at the school and consisted of two parts following directly after each other. In the first part, children had to position 20 numbers that were randomly selected with the following restrictions: (a) two numbers from each hundred between 0 and 1,000 were selected to ensure a more or less equal distribution of the numbers across the number line, (b) eight of these numbers (underlined, cf. infra) were chosen in the immediate neighborhood of 0, 250, 500, 750, and 1,000 to detect the possible use of benchmarks by means of a contour analysis (Ashcraft and Moore, 2012), and (c) every unit, decade, and hundred appeared exactly two times. The twenty target numbers were: 7, 61, 123, 170, 246, 254, 310, 386, 439, 498, 502, 521, 613, 687, 742, 758, 839, 875, 965, and 993. The presentation of the numbers was randomized across participants. To get familiarized with the task and the procedure, children also received two practice numbers containing the target numbers 196 and 633. Children were given the following instruction: “I am going to show you some number lines. These number lines start at 0 and end at 1,000. For each number line, I am going to present you a card with a number between 0 and 1,000. I want you to put a mark on the number line where you think the number would go.” The target numbers were read out loud by the experimenter to ensure that children knew which number had to be positioned on the number line.

In the second part we relied on verbal trial-by-trial strategy reports as an additional data source, to assess whether children used benchmarks when solving the task. At the end of the task, children had to estimate the position of five additional target numbers that were located close to the different benchmarks: 3, 251, 506, 745, and 997. Immediately after each estimate, the experimenter asked the child to explain how (s)he came to that estimate with questions like: “How did you do come to that estimate?” and “What were you thinking?” Children’s verbal responses were recorded by a voice recorder. Total completion time of the NLE task took on average about 15 min per child. Feedback in terms of repeating the task instruction was only provided when participants interpreted the task incorrectly (for instance, when they made estimates based on a 0–100 number line).

Finally, third and sixth graders’ mathematics achievement was measured by means of the standardized mathematics test of the Flemish Student Monitoring System (Dudal, 2000) for the beginning of the third and sixth grade, respectively. These test were already administered by the schoolteachers as part of the school’s regular evaluation system.

### Strategy Classification

**FIGURE 2:**
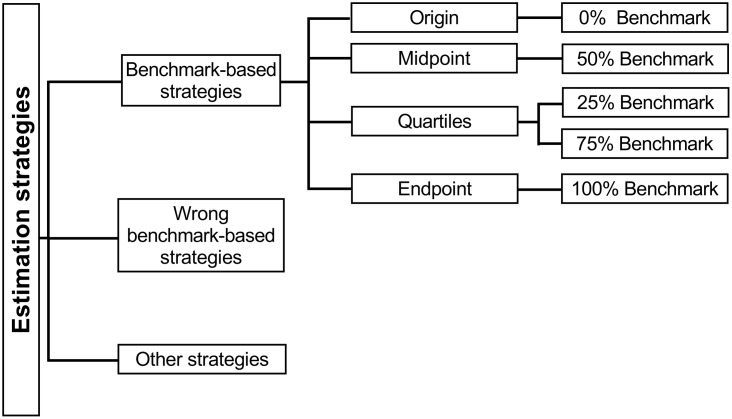
Decision tree for the classification of NLE strategies.

Strategy reports were analyzed by means of a classification scheme that was slightly adapted from Peeters et al. (2017), consisting of three main categories: benchmark-based strategies, wrong benchmark-based strategies, and other strategies (**Figure 2**). The benchmark-based strategy category consisted of strategies based on an externally provided or internal (i.e., self-generated) benchmark located at the origin (0%), the endpoint (100%), the midpoint (50%), and the quartiles (25 and 75%) of the number line. Strategies referring to more than one benchmark (e.g., I looked at 0 and 250, and then I placed 170 left from 250), were coded as relying on the most sophisticated benchmark (i.e., quartiles > midpoint > endpoint > origin). The second category contained wrong benchmark-based strategies. These are strategies based on incorrectly identified benchmarks. For example, a child who places his/her mark after the first hatch mark on the unlabeled number line and explains that “the first hatch mark is 260, so I should place 251 before the mark.” This child wrongly associates the first benchmark with 260 instead of 250 and based on this knowledge (s)he places the number 251 before the first benchmark, instead of right after it. The third category, called the other strategies category, included (a) all strategies that did not belong to the first nor second category, such as a strategy based on the estimate of the target number of a previous trial (e.g., “A moment ago, I placed 687 about here, so 613 is a bit to the left”; see also Sullivan and Barner, 2014), and (b) cases wherein no clear strategy could be derived from the child’s verbal protocol (e.g., “This piece is about 2 cm”).

Reliability of the classification scheme was assessed by testing the agreement in classification of two independent raters who classified all verbal strategy reports of nine randomly chosen third graders and nine randomly chosen sixth graders (three from each of the three conditions). This resulted in a Cohen’s Kappa of 0.91 for the third graders and 0.86 for the sixth graders, both indicating almost perfect agreement according to the standards of Landis and Koch (1977).

## Results

### Estimation Accuracy

To determine the accuracy of children’s NLEs in general and around the benchmarks in particular, we first measured the distance from the origin of the number line to their handwritten mark. Next, the measured distance was converted into a numerical estimate. We then calculated the Percentage of Absolute Error (PAE) for each of the numerical estimates. Individual estimates that deviated more than 2 *SD*s from children’s mean estimate were excluded. For the third graders, 82 estimates out of a total of 1660 were removed (i.e., 5%, of which 31 in the control condition, 27 in the unlabeled condition, and 24 in the labeled condition). For the sixth graders, 77 estimates out of a total of 1440 were removed (i.e., 5%, of which 23 in the control condition, 28 in the unlabeled condition, and 26 in the labeled condition). Tukey HSD tests were used for all *post hoc* comparisons.

#### Overall Estimation Accuracy

**FIGURE 3:**
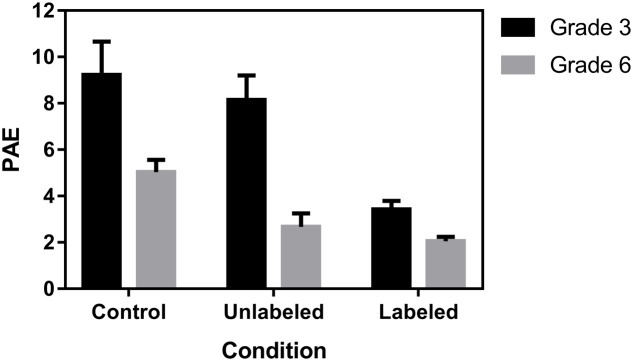
Mean percentage of absolute error (PAE and 95% CI) as a function of grade and condition.

A 3 (Condition: control, unlabeled, and labeled) × 2 (Grade: 3 vs. 6) ANOVA on PAE showed a significant interaction effect, *F*(2,149) = 12.85, *p* < 0.0001 (**Figure 3**). As expected, the estimates of the third graders were significantly more accurate in the labeled condition (*M* = 3%, *SD* = 2%) in comparison to the unlabeled (*M* = 8%, *SD* = 7%, *p* < 0.0001) and control condition (*M* = 9%, *SD* = 8%, *p* < 0.0001). Sixth graders, on the other hand, made more accurate estimates in the labeled (*M* = 2%, *SD* = 2%, *p* < 0.0001) and unlabeled condition (*M* = 3%, *SD* = 3%, *p* = 0.001) in comparison to the control condition (*M* = 5%, *SD* = 4%). Neither the difference in overall accuracy between unlabeled and control conditions in third graders nor the difference in overall accuracy between labeled and unlabeled conditions in sixth graders was significant.

#### Estimation Accuracy around the Benchmarks

**FIGURE 4:**
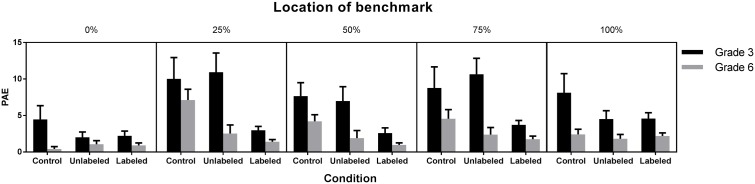
Mean PAE (and 95% CI) at the benchmarks as a function of grade and condition.

To investigate the estimation accuracy around the benchmarks, a contour analysis (Ashcraft and Moore, 2012) was conducted. Therefore, we averaged, on a child-by-child basis, the observed PAE for the two target numbers located immediately before and after 250, 500, and 750 (i.e., the numbers for which an additional external benchmark was presented in the unlabeled and labeled condition). For the origin and endpoint, our measure was based on the PAE of the number immediately after the origin and immediately before the endpoint, respectively. A 3 (Condition: control, unlabeled, labeled) × 2 (Grade: 3 vs. 6) × 5 (Location of benchmark: 0, 250, 500, 750, 1,000) ANOVA with repeated measures on the last variable and PAE as dependent variable revealed a significant three-way interaction between condition, grade, and location, F(8,580) = 5.56, p < 0.0001 (**Figure 4**).

The estimates of the third and sixth graders showed no significant differences across conditions around the origin and endpoint. Third graders estimated near benchmarks 250, 500 and 750 more accurately in the labeled condition (*M*_250_ = 3%, *SD*_250_ = 1%; *M*_500_ = 3%, *SD*_500_ = 2%; *M*_750_ = 4%, *SD*_750_ = 2%) than in the unlabeled (*M*_250_ = 11%, *SD*_250_ = 7%, *p*_250_ < 0.0001; *M*_500_ = 7%, *SD*_500_ = 5%, *p*_500_ = 0.001; *M*_750_ = 11%, *SD*_750_ = 6%, *p*_750_ < 0.0001) and control condition (*M*_250_ = 10%, *SD*_250_ = 7%, *p*_250_ < 0.0001; *M*_500_ = 8%, *SD*_500_ = 5%, *p*_500_ < 0.0001; *M*_750_ = 9%, *SD*_750_ = 7%, *p*_750_ < 0.0001). The sixth graders made, near benchmark 250, more accurate estimates in the labeled (*M* = 1%, *SD* = 1%, *p <* 0.0001) and unlabeled condition (*M* = 3%, *SD* = 3%, *p <* 0.0001) in comparison to the control condition (*M* = 7%, *SD* = 4%). Their estimates showed no differences among conditions around benchmark 500 and 750 (*p*s > 0.10).

### Strategy Use

#### Strategy Categories

Firstly, we analyzed how often children used a strategy from one of the three categories of the above-mentioned classification scheme by conducting a 3 (Condition: control, unlabeled, labeled) × 2 (Grade: 3 vs. 6) × 3 (Category: benchmark-based, wrong benchmark-based, other) ANOVA with repeated measures on the last variable and the percentage of target numbers being solved with a specific strategy as dependent variable. This revealed a significant interaction between condition, grade, and category, *F*(4,298) = 7,87, *p* < 0.0001. There were no differences among conditions regarding the use of benchmark-based, other or wrong benchmark-based strategies in sixth graders (*p*s > 0.10, **Table 1**). Third graders, however, applied benchmark-based strategies more often in the labeled (*M* = 96%, *SD* = 12%, *p* = 0.01) and control condition (*M* = 85%, *SD* = 20%, *p* < 0.0001) than in the unlabeled condition (*M* = 72%, *SD* = 23%). At first sight, it seems that third graders are applying benchmark-based strategies less frequently in the unlabeled than in the control condition. However, when adding up the percentages of the benchmark-based and wrong benchmark-based category, it turns out that third graders use (correct and wrong) benchmark-based strategies more frequently in the unlabeled (96%) than in the control condition (86%). This finding suggests that the additional external benchmarks in the unlabeled condition elicited benchmark-based strategies in third graders but that the benchmarks are not always identified correctly. Overall, these results show that children in both grades use benchmark-based strategies on the majority of the trials. Therefore, the remainder of the strategy analyses zoomed in on this category of NLE strategies.

**Table 1:** Percentage of trials solved by benchmark-based, wrong benchmark-based, and other strategies for third and sixth graders in the control, unlabeled, and labeled condition.

	Grade 3			Grade 6		
	Control	Unlabeled	Labeled	Control	Unlabeled	Labeled
Benchmark-based	85 (20)	72 (23)	96 (12)	94 (9)	97 (10)	100 (0)
Other	14 (20)	4 (13)	3 (12)	5 (9)	1 (6)	0 (0)
Wrong benchmark-based	1 (4)	24 (22)	1 (4)	1 (4)	2 (6)	0 (0)

*Standard deviations appear in parentheses*.

#### Variety of Benchmark-Based Strategies

**FIGURE 5:**
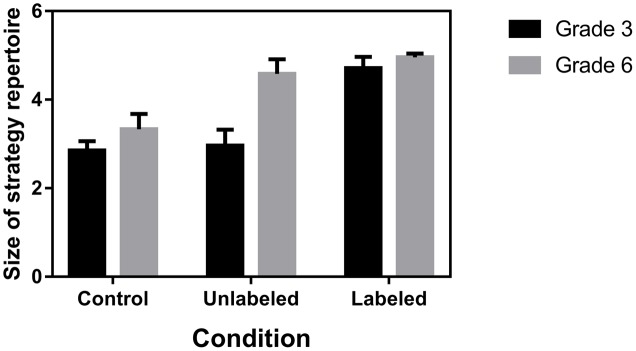
Number of benchmark-based strategies in children’s strategy repertoire (with 95% CI) as a function of grade and condition.

The effect of the provision of the additional external benchmarks and labeling of these benchmarks on the size of children’s strategy repertoire was investigated. The size of children’s strategy repertoire was measured by calculating the number of different strategies that were based on the benchmarks 0, 250, 500, 750, and 1,000 in the second part of the NLE task, with a maximum of five strategies for each child. A 3 (Condition: control, unlabeled, labeled) × 2 (Grade: 3 vs. 6) ANOVA with size of strategy repertoire as dependent variable, showed a significant interaction effect, *F*(2,149) = 13.91, *p* < 0.0001 (**Figure 5**). In accordance with our hypothesis, third graders had a significantly larger strategy repertoire in the labeled condition (*M* = 4.71, *SD* = 0.64), in comparison to the unlabeled (*M* = 2.96, *SD* = 0.91, *p* < 0.0001) and control condition (*M* = 2.85, *SD* = 0.52, *p* < 0.0001), whereas the strategy repertoire of sixth graders was significantly larger in both the labeled (*M* = 4.96, *SD* = 0.20, *p* < 0.0001) and unlabeled condition (*M* = 4.58, *SD* = 0.76, *p* < 0.0001) than in the control condition (*M* = 3.33, *SD* = 0.80).

#### Frequency of Benchmark-Based Strategies

**FIGURE 6:**
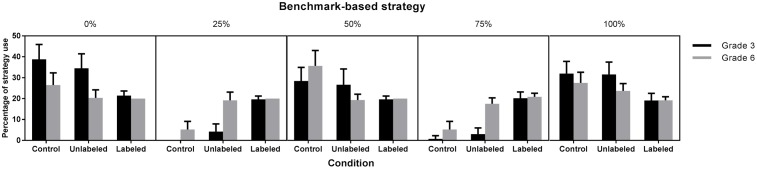
Frequency of the benchmark-based strategies (with 95% CI) as a function of grade and condition.

Based on children’s verbal reports, we determined the percentage of trials that was solved using one of the benchmark-based strategies. A 3 (Condition: control, unlabeled, labeled) × 2 (Grade: 3 vs. 6) × 5 (Benchmark: 0, 250, 500, 750, 1,000) ANOVA with repeated measures on the last variable and the percentage of trials solved using one of the benchmarks as dependent variable, showed a significant three-way interaction, *F*(8,596) = 5.14, p < 0.0001 (**Figure 6**).

When considering the results of the third graders, we observed that more of them used the origin (0) and endpoint (1,000) in the control (*M*_0_ = 39%, *SD*_0_ = 18%, *p*_0_ < 0.0001; *M*_1,000_ = 32%, *SD*_1,000_ = 15%, *p*_1,000_ = 0.002) and unlabeled condition (*M*_0_ = 35%, *SD*_0_ = 18%, *p*_0_ = 0.0014; *M*_1,000_ = 32%, *SD*_1,000_ = 15%, *p*_1,000_ = 0.004) than in the labeled condition (*M*_0_ = 21%, *SD*_0_ = 6%; *M*_1,000_ = 19%, *SD*_1,000_ = 9%). Furthermore, there were no differences among conditions for the use of the midpoint (*p*s > 0.10). As for the quartile benchmarks (i.e., 250 and 750), we observed that, as expected, more third graders used them in the labeled condition (*M*_250_ = 20%, *SD*_250_ = 4%; *M*_750_ = 20%, *SD*_750_ = 8%) compared to the unlabeled (*M*_250_ = 4%, *SD*_250_ = 10%, *p*_250_ < 0.0001; *M*_750_ = 3%, *SD*_750_ = 8%, *p*_750_ < 0.0001) or control condition (*M*_250_ = 0%, *SD*_250_ = 0%, *p*_250_ < 0.0001; *M*_750_ = 1%, *SD*_750_ = 4%, *p*_750_ < 0.0001), suggesting that the less frequent use of the origin and endpoint in the labeled condition was compensated by a more frequent use of the quartile benchmarks in that condition. When looking at the sixth graders, we found no differences among conditions in the use of the origin and endpoint. Moreover, more sixth graders made use of the midpoint in the control condition (*M* = 36%, *SD* = 18%) than in the unlabeled (*M* = 19%, *SD* = 7%, *p* < 0.0001) or labeled condition (*M* = 20%, *SD* = 0%, *p* = 0.0001). In contrast, the quartile benchmarks (i.e., 250 and 750) were used by more sixth graders in the labeled (*M*_250_ = 20%, *SD*_250_ = 0%, *p*_250_ = 0.0005; *M*_750_ = 21%, *SD*_750_ = 4%, *p*_750_ = 0.0001) and unlabeled condition (*M*_250_ = 19%, *SD*_250_ = 9%, *p*_250_ = 0.002; *M*_750_ = 18%, *SD*_750_ = 7%, *p*_750_ = 0.018) than in the control condition (*M*_250_ = 5%, *SD*_250_ = 9%; *M*_750_ = 5%, *SD*_750_ = 9%), which confirms our hypothesis.

### Relationship between Estimation Accuracy, Benchmark-Based Strategy Use, and Mathematics Achievement

Partial correlations, controlling for both grade and condition, were used to investigate the relationship between PAE, the variety of benchmarks that children used, the frequency with which they applied quartile-based strategies and their mathematics achievement (**Table 2**). We observed, in line with our prediction, a negative correlation between PAE, the size of children’s strategy repertoire, and the frequency with which they used quartile-based strategies. This indicates that a more accurate performance was related to a larger variety of strategies and a more frequent use of quartile-based strategies. Furthermore, we can confirm our hypothesis that mathematics achievement is positively related to children’s estimation accuracy, strategy repertoire, and quartile-based strategy use.

**Table 2:** Partial correlations controlling for grade and condition.

Variable	1	2	3
(1) PAE	–		
(2) Strategy repertoire	-0.28^∗∗^	–	
(3) % quartile-based strategies	-0.29^∗∗∗^	0.72^∗∗∗^	–
(4) Mathematics achievement	-0.21^∗∗^	0.21^∗∗^	0.27^∗∗^

^∗∗^*p < 0.01; ^∗∗∗^*p* < 0.001*.

## Discussion

According to the log-to-lin account, age-related improvements in NLE performance are the result of changes in children’s mental magnitude representation (Siegler and Opfer, 2003; Siegler and Booth, 2004; Booth and Siegler, 2006; Laski and Siegler, 2007). Recently, however, various kinds of evidence indicate that changes in children’s benchmark-based strategy use might also explain the observed developments in their NLE performance (e.g., Newman and Berger, 1984; Petitto, 1990; Barth and Paladino, 2011; Ashcraft and Moore, 2012; White and Szucs, 2012; Slusser et al., 2013; Rouder and Geary, 2014; Peeters et al., 2017, unpublished). More specifically, these studies revealed that participants solve the NLE task by applying strategies based on benchmarks at the origin, midpoint, and endpoint of the number line. Moreover, Peeters et al. (2017) recently demonstrated that adults rely on strategies based on internal benchmarks at the quartiles (i.e., 25 and 75%) of the number line too. The results of a follow-up study on children indicated that, in contrast to fifth graders, third graders’ overall estimation accuracy and frequency of quartile-based strategy use did not increase when more benchmark support was given on the number line (Peeters et al., unpublished).

Importantly, numerical values were not provided for the additional external benchmarks at 25, 50, and 75% of the number line and the authors argued that third graders’ use of quartile-based strategies in the quartile condition might not have increased due to their inability to link the corresponding numerical values of 250, 500, and 750 to the additional external benchmarks at 25, 50, and 75%, respectively. Therefore, the main aim of the present study was to investigate whether third and sixth graders’ estimation performance and quartile-based strategy use on a 0–1,000 number line would improve if the corresponding numerical values underneath the external benchmarks were provided. Moreover, we examined whether the potential positive effect of labeling was dependent on children’s grade. To achieve these goals, we asked third and sixth graders to solve a 0–1,000 NLE task. The degree of external benchmark support was manipulated in three separate conditions: a control condition bounded by 0 at the origin and 1,000 at the endpoint, an unlabeled condition with three additional external benchmarks at 25, 50, and 75% of the number line but without numerical labels, and a labeled condition with numerical labels underneath all external benchmarks.

Our results indicate that the effect of labeling the benchmarks was, as expected, dependent on children’s age. As evidenced by third graders’ better NLE performance in the labeled than in the unlabeled and control condition, labeling the benchmarks was necessary for improving their estimation accuracy. On the other hand, sixth graders’ NLE performance showed an equally strong improvement in both the unlabeled and labeled condition in comparison with the control condition, indicating that the mere presentation of external benchmarks at 25, 50, and 75% of the number line was already sufficient for improving their accuracy. A similar pattern of results was observed for third and sixth graders’ estimation accuracy in the immediate neighborhood of the additional external benchmarks, suggesting that third graders could only use the midpoint- and quartile-based strategies when the benchmarks were labeled, whereas sixth graders could already rely on these strategies when confronted with unlabeled benchmarks.

The findings from the contour analysis were confirmed by the strategy results. More specifically, labeling the benchmarks had a beneficial effect on third graders’ strategy use: they did not only use a greater variety of benchmark-based strategies but used more quartile-based strategies in the labeled condition compared to the unlabeled and control condition. Sixth graders’ greater variety of benchmark-based strategies and more frequent application of quartile-based strategies in both the unlabeled and labeled condition compared to the control condition, indicated that the mere presentation of external benchmarks at 25, 50, and 75% of the number line were already sufficient to obtain a positive effect and labeling the additional external benchmarks had no further beneficial effect on their strategy use.

Confirming previous results (e.g., Ashcraft and Moore, 2012; White and Szucs, 2012; Peeters et al., unpublished), our findings indicate that third and sixth graders spontaneously generate and use an internal benchmark at the midpoint. Although sixth graders used the midpoint to a lesser extent in the unlabeled and labeled condition, this was compensated by a more frequent application of quartile-based strategies in those conditions. This result suggests that sixth graders choose their strategies more adaptively in the labeled condition than third graders (Lemaire and Siegler, 1995). Furthermore, our results indicate that children’s use of this refined quartile-based strategy use can be stimulated by adding additional external benchmarks on the number line. Depending on children’s age and familiarity with the number range, however, these additional external benchmarks might need to be labeled. Overall, these findings are in line with the view that developmental changes in NLE performance are due to refinements in children’s benchmark-based strategy use, rather than to a shift in their mental magnitude representation from logarithmically to linearly shaped.

Also in agreement with earlier findings is the observation that estimation accuracy is positively associated with mathematics achievement (e.g., Siegler and Booth, 2004; Booth and Siegler, 2006, 2008; Geary et al., 2007; Laski and Siegler, 2007; Sasanguie et al., 2012). Moreover, having a broader strategy repertoire was positively related with children’s estimation accuracy, as was the case in Lemaire and Arnaud’s (2008) study where errors on complex addition problems decreased as more strategies were incorporated into a person’s strategy repertoire. Also, applying quartile-based strategies more frequently was positively associated with children’s estimation accuracy as well as with their mathematics achievement (Peeters et al., 2017, unpublished). The relationship with mathematics achievement suggests that children who are more proficient in mathematics may be more prone to generate more refined benchmarks on the number line, allowing them to make more accurate estimates. This finding is in line with Link et al. (2014b) who suggested that the creation of such benchmarks is based on diverse components of children’s mathematical knowledge and skills, such as their proportional reasoning ability, number comparison skills, and arithmetic proficiency. These components allow them to generate internal benchmarks at the midpoint or quartiles by dividing the number line into two or into four, respectively (e.g., 250 is halfway 0 and 500), to decide through number comparison whether the target number is smaller or larger than the numerical value of a particular benchmark (e.g., knowing that 243 is located before 250), and to further refine the estimated position of the target number in relation to the benchmark by means of addition or subtraction (e.g., 250-7 is 243). The suggestion that children’s NLE performance draws on several components of their mathematical knowledge and skills, including their numerical magnitude knowledge, could explain the observed association between children’s NLE performance and their scores on standardized mathematics achievement tests.

Taking into account this association, a possible avenue for future research would be examining whether training children to use quartile-based strategies would have an effect on their NLE performance, and perhaps more importantly, on their proportional reasoning skills and their ability to add, subtract, and compare numbers, which might in its turn positively affect their overall mathematical competence. Training children to use quartile-based strategies can be achieved by offering them additional external benchmarks at 25, 50, and 75% of the number line. However, as suggested by the present findings, one should take into account that, depending on children’s age and familiarity with the number range, these additional external benchmarks need to be labeled. It might also be interesting to know whether this kind of training would have a stronger beneficial effect on children’s arithmetic skills and mathematics achievement compared to playing linear board games which mainly focuses on stimulating the linear representation of numerical magnitudes (e.g., Whyte and Bull, 2008; Siegler and Ramani, 2009; Ramani et al., 2012).

Another possible avenue for future research would be to investigate the extent to which decile (i.e., at every 10% of the number line) or quintile (i.e., at every 20% of the number line) benchmarks might be helpful for children. A future study could provide children with either an empty number line or a number line containing quartile, quintile, or decile benchmarks. This approach would allow investigating which kind of benchmark support (quartile, quintile, or decile) is most beneficial and whether this would be different, depending on children’s age.

Finally, it may also be interesting – particularly from an educational perspective – to design, implement, and test (computer-supported) learning environments that optimally help learners to make the transition from making NLEs by relying on externally provided benchmarks to making appropriate use of self-generated internal benchmarks. Designers of such learning environments could rely on models for instructional design, such as Collins et al. (1989) well-grounded “cognitive apprenticeship” model, wherein complex cognitive skills are built, among others, by first providing the learners with external supporting tools that help them to perform the given cognitive task properly (=, ‘scaffolding’; e.g., providing external benchmarks and their corresponding numerical labels) and that are gradually taken away (= ‘fading,’ e.g., removing the numerical labels first and the external benchmarks afterward) until they can perform the task independently.

## Ethics Statement

This study was carried out in accordance with the institutional ethical guidelines of KU Leuven. At the moment the data were collected (i.e., February/March 2014), the ethical review board (SMEC) which is responsible for the ethical review of research in the behavioral sciences was not founded yet.

## Author Contributions

All authors contributed extensively to the work presented in this paper. DP, ES, LV, and KL designed the experiment. ES conducted the experiment. DP and ES analyzed the data and wrote the first draft of the paper. LV and KL supervised the analyses and assisted in the revision and finalization of the paper. The authors would like to thank Sarah Ruys-Van Krunkelsven for her help in data collection.

## Conflict of Interest Statement

The authors declare that the research was conducted in the absence of any commercial or financial relationships that could be construed as a potential conflict of interest.
